# Interhospital Transfer Before Thrombectomy Is Associated With Delayed Treatment and Worse Outcome in the STRATIS Registry (Systematic Evaluation of Patients Treated With Neurothrombectomy Devices for Acute Ischemic Stroke)

**DOI:** 10.1161/CIRCULATIONAHA.117.028920

**Published:** 2017-12-11

**Authors:** Michael T. Froehler, Jeffrey L. Saver, Osama O. Zaidat, Reza Jahan, Mohammad Ali Aziz-Sultan, Richard P. Klucznik, Diogo C. Haussen, Frank R. Hellinger, Dileep R. Yavagal, Tom L. Yao, David S. Liebeskind, Ashutosh P. Jadhav, Rishi Gupta, Ameer E. Hassan, Coleman O. Martin, Hormozd Bozorgchami, Ritesh Kaushal, Raul G. Nogueira, Ravi H. Gandhi, Eric C. Peterson, Shervin R. Dashti, Curtis A. Given, Brijesh P. Mehta, Vivek Deshmukh, Sidney Starkman, Italo Linfante, Scott H. McPherson, Peter Kvamme, Thomas J. Grobelny, Muhammad S. Hussain, Ike Thacker, Nirav Vora, Peng Roc Chen, Stephen J. Monteith, Robert D. Ecker, Clemens M. Schirmer, Eric Sauvageau, Alex Abou-Chebl, Colin P. Derdeyn, Lucian Maidan, Aamir Badruddin, Adnan H. Siddiqui, Travis M. Dumont, Abdulnasser Alhajeri, M. Asif Taqi, Khaled Asi, Jeffrey Carpenter, Alan Boulos, Gaurav Jindal, Ajit S. Puri, Rohan Chitale, Eric M. Deshaies, David H. Robinson, David F. Kallmes, Blaise W. Baxter, Mouhammad A. Jumaa, Peter Sunenshine, Aniel Majjhoo, Joey D. English, Shuichi Suzuki, Richard D. Fessler, Josser E. Delgado Almandoz, Jerry C. Martin, Nils H. Mueller-Kronast

**Affiliations:** 1Vanderbilt University Medical Center, Nashville, TN (M.T.F., R.C.).; 2University of California, Los Angeles (J.L.S., R.J., D.S.L., S.S.).; 3St Vincent Mercy Hospital, Toledo, OH (O.O.Z.).; 4Brigham and Women’s Hospital, Boston, MA (M.A.A.-S.).; 5Methodist Hospital, Houston, TX (R.P.K.).; 6Emory University School of Medicine, Grady Memorial Hospital, Atlanta, GA (D.C.H., R.G.N.).; 7Florida Hospital Neuroscience Institute, Winter Park (F.R.H., R.H.G.).; 8University of Miami Miller School of Medicine/Jackson Memorial Hospital, FL (D.R.Y., E.C.P.).; 9Norton Neuroscience Institute, Norton Healthcare, Louisville, KY (T.L.Y., S.R.D.).; 10University of Pittsburgh Medical Center, PA (A.P.J.).; 11WellStar Neurosciences Network, WellStar Kennestone Regional Medical Center, Marietta, GA (R.G.).; 12Valley Baptist Medical Center, Harlingen, TX (A.E.H.).; 13St. Luke’s Hospital of Kansas City, MO (C.O.M.).; 14Oregon Health and Science University Hospital, Portland (H.B.).; 15Advanced Neuroscience Network/Tenet South Florida, Delray Beach (R.K., N.H.M.-K.).; 16Baptist Health Lexington/Central Baptist, KY (C.A.G.).; 17South Broward Hospital, Hollywood, FL (B.P.M.).; 18Providence St Vincent Medical Center, Portland, OR (V.D.).; 19Baptist Hospital of Miami, FL (I.L.).; 20St Dominic’s-Jackson Memorial Hospital, MS (S.H.M.).; 21University of Tennessee Medical Center, Knoxville (P.K.).; 22Advocate Christ Medical Center, Oak Lawn, IL (T.J.G.).; 23Cleveland Clinic, OH (M.S.H.).; 24Baylor University Medical Center, Dallas, TX (I.T.).; 25OhioHealth Riverside Methodist Hospital, Columbus (N.V.).; 26Memorial Hermann Texas Medical Center, Houston (P.R.C.).; 27Swedish Medical Center First Hill Campus, Seattle, WA (S.J.M.).; 28Maine Medical Center, Portland (R.D.E.).; 29Geisinger Clinic, Danville, PA (C.M.S.).; 30Baptist Medical Center–Jacksonville, FL (E.S.).; 31Baptist Hospital Louisville, KY (A.A.-C.).; 32Barnes Jewish Hospital, St Louis, MO (C.P.D.).; 33Mercy San Juan Medical Center and Mercy General, Carmichael, CA (L.M.).; 34Presence St Joseph Medical Center, Joliet, IL (A.B.).; 35Buffalo General Medical Center, NY (A.H.S.).; 36University of Arizona Medical Center, Tucson (T.M.D.).; 37University of Kentucky Hospital, Lexington (A.A.).; 38Los Robles Medical Center, Thousand Oaks, CA (M.A.T.).; 39Aurora Hospital, Milwaukee, WI (K.A.).; 40West Virginia University/Ruby Memorial Hospital, Morgantown (J.C.).; 41Albany Medical Center, NY (A.B.).; 42University of Maryland Medical Center, Baltimore (G.J.).; 43University of Massachusetts Memorial Medical Center, Worcester (A.S.P.).; 44Crouse Hospital, Syracuse, NY (E.M.D.).; 45Virginia Mason Medical Center, Seattle, WA (D.H.R.).; 46Mayo Clinic–Rochester, MN (D.F.K.).; 47Erlanger Medical Center, Chattanooga, TN (B.W.B.).; 48ProMedica Toledo Hospital, OH (M.A.J.).; 49Banner University Medical Center, Phoenix, AZ (P.S.).; 50McLaren Flint, MI (A.M.).; 51California Pacific Medical Center, San Francisco, CA (J.D.E.).; 52University of California, Irvine, Orange (S.S.).; 53St John Providence Hosptial, Detroit, MI (R.D.F.).; 54Abbott Northwestern Hospital, Minneapolis, MN (J.E.D.A.). Carolinas Medical Center, Charlotte, NC.

**Keywords:** emergency medical services, endovascular treatment, ischemic stroke, stent retriever, systems of care

## Abstract

**Background:**

Endovascular treatment with mechanical thrombectomy (MT) is beneficial for patients with acute stroke suffering a large-vessel occlusion, although treatment efficacy is highly time-dependent. We hypothesized that interhospital transfer to endovascular-capable centers would result in treatment delays and worse clinical outcomes compared with direct presentation.

**Methods:**

STRATIS (Systematic Evaluation of Patients Treated With Neurothrombectomy Devices for Acute Ischemic Stroke) was a prospective, multicenter, observational, single-arm study of real-world MT for acute stroke because of anterior-circulation large-vessel occlusion performed at 55 sites over 2 years, including 1000 patients with severe stroke and treated within 8 hours. Patients underwent MT with or without intravenous tissue plasminogen activator and were admitted to endovascular-capable centers via either interhospital transfer or direct presentation. The primary clinical outcome was functional independence (modified Rankin Score 0–2) at 90 days. We assessed (1) real-world time metrics of stroke care delivery, (2) outcome differences between direct and transfer patients undergoing MT, and (3) the potential impact of local hospital bypass.

**Results:**

A total of 984 patients were analyzed. Median onset-to-revascularization time was 202.0 minutes for direct versus 311.5 minutes for transfer patients (*P*<0.001). Clinical outcomes were better in the direct group, with 60.0% (299/498) achieving functional independence compared with 52.2% (213/408) in the transfer group (odds ratio, 1.38; 95% confidence interval, 1.06–1.79; *P*=0.02). Likewise, excellent outcome (modified Rankin Score 0–1) was achieved in 47.4% (236/498) of direct patients versus 38.0% (155/408) of transfer patients (odds ratio, 1.47; 95% confidence interval, 1.13–1.92; *P*=0.005). Mortality did not differ between the 2 groups (15.1% for direct, 13.7% for transfer; *P*=0.55). Intravenous tissue plasminogen activator did not impact outcomes. Hypothetical bypass modeling for all transferred patients suggested that intravenous tissue plasminogen activator would be delayed by 12 minutes, but MT would be performed 91 minutes sooner if patients were routed directly to endovascular-capable centers. If bypass is limited to a 20-mile radius from onset, then intravenous tissue plasminogen activator would be delayed by 7 minutes and MT performed 94 minutes earlier.

**Conclusions:**

In this large, real-world study, interhospital transfer was associated with significant treatment delays and lower chance of good outcome. Strategies to facilitate more rapid identification of large-vessel occlusion and direct routing to endovascular-capable centers for patients with severe stroke may improve outcomes.

**Clinical Trial Registration:**

URL: https://www.clinicaltrials.gov. Unique identifier: NCT02239640.

## Abstract 2

Supplemental Digital Content is available in the text.

**Editorial, see p 2322**

**Clinical Perspective**

**What Is New?**

- This large real-world study shows that patients who are transferred between hospitals for endovascular stroke treatment have worse outcomes compared with those who presented directly to an endovascular-capable center.
- Delays to endovascular treatment account for the difference in outcomes between direct and transfer patients.
- A hypothetical bypass analysis showed that if patients were brought directly to the endovascular-capable center, tissue plasminogen activator would be slightly delayed by 12 minutes, but endovascular treatment would be delivered 91 minutes sooner.
- If limited to bypass of <20 miles, tissue plasminogen activator is delayed by only 7 minutes, but endovascular treatment is delivered 94 minutes sooner.

**What Are the Clinical Implications?**

- These findings reinforce that early recanalization leads to better outcomes. Stroke systems of care should be optimized to get patients to appropriate treatment quickly.
- When possible, interhospital transfer should be avoided.
- Our hypothetical bypass analysis showed that transporting patients directly to endovascular-capable facilities has only a modest impact on timing of intravenous tissue plasminogen activator delivery but can have a significant reduction in time to endovascular recanalization.
- This finding was especially true for bypass distances of <20 miles.
- Thus, bypass to the nearest endovascular-capable stroke center should be strongly considered if it is within 20 miles.

Ischemic stroke is the leading cause of disability in the United States, and the burden of stroke continues to increase worldwide.^1,2^ Acute large-vessel occlusion (LVO) causes some of the largest and most disabling strokes.^3^ Fortunately, multiple recent trials have shown that endovascular mechanical thrombectomy (MT) leads to significantly better outcomes in patients with LVO stroke compared with medical therapy alone.^4–8^ STRATIS (Systematic Evaluation of Patients Treated With Neurothrombectomy Devices for Acute Ischemic Stroke) was a prospective, multicenter, observational, single-arm registry of patients with LVO stroke treated with MT, which showed that outcomes similar to those observed in clinical trials can be achieved in a real-world setting.^9^

The strongly positive effect of MT in acute stroke is highly time-dependent. A recent patient-level meta-analysis of MT for LVO showed that the benefit associated with MT decreases with each 1-hour delay to treatment, and that benefit became nonsignificant after 7.3 hours from stroke onset.^10^ Thus, achieving timely treatment is critically important to realize the benefits of MT. This timeliness is highly dependent on the entire system of care, including prehospital triage and routing. Many patients with acute stroke are transported to the nearest hospital, which is often not capable of endovascular treatment. In these cases, patients undergo a rapid diagnostic workup, and may receive intravenous tissue-type plasminogen activator (IV-tPA) at the community hospital before being transferred to an endovascular-capable center. We hypothesized that such transfers might be associated with additional treatment delays and consequent reduction in likelihood of good clinical outcome.

## Methods

### Study Design and Inclusion Criteria

STRATIS was a prospective, multicenter, observational registry evaluating the use of Medtronic market-released MT devices in consecutive patients with acute ischemic stroke because of LVO in the anterior circulation between August 2014 and June 2016 from 55 sites (see Table I in the online-only Data Supplement for individual site characteristics) with approval from local institutional review boards. Patients were enrolled from the time of arrival at the enrolling hospital up to the day of hospital discharge, or ≤7 days after MT. Enrollment was planned to include ≤1000 patients to create the largest registry of patients with LVO stroke to date.

The inclusion criteria were (1) informed consent before enrollment in the registry, (2) acute stroke because of LVO that had been or would be treated with a Medtronic market-released MT device as the initial device, (3) treatment within 8 hours of stroke onset, (4) prestroke modified Rankin Scale (mRS) score of ≤2, and (5) pretreatment National Institutes of Health Stroke Scale (NIHSS) score of ≥8 and ≤30 as reported by the enrolling center. The qualifying NIHSS could be collected at either the initial or enrolling hospital, and there was no requirement that it be repeated before MT. Use of a Medtronic MT device and enrollment in the registry were at the discretion of the site clinician-investigator; patients treated with other devices were not included in the registry.

### Outcomes

The primary outcome was functional status as assessed by mRS at 90 days performed by the local treatment team, either in person or by telephone, and verification of assessor certification was not verified. Clinical end points were defined as follows: mRS 0 to 2 = functional independence, and mRS 0 to 1 = excellent clinical outcome. Total stroke onset-to-revascularization time was a secondary end point. Revascularization was defined as modified Thombolysis in Cerebral Ischemia 2b or 3, corresponding to substantial or complete reperfusion, and was determined by an independent core imaging laboratory. Only patients achieving successful revascularization were included in time analyses. Patients were compared between 2 groups: those who presented directly to the endovascular-capable hospital (direct) versus patients who came by interhospital transfer to the endovascular-capable hospital (transfer) and were additionally categorized as having received IV-tPA before MT or undergoing MT alone. The impacts of time to treatment, direct presentation versus transfer, and IV-tPA were assessed with a multivariate logistic regression accounting for other covariates. For analyses of outcome and time, the onset-to-puncture time epoch was used rather than time to revascularization so that all patients were included. Mortality rates at 90 days were also compared between direct and transfer groups. Early ischemic changes on the initial noncontrast head computed tomography were quantified using the ASPECTS score (Alberta Stroke Program Early CT Score), a validated measure of ischemic changes correlating with clinical outcome.^11^ All imaging was evaluated by a core laboratory (University of California, Los Angeles Neurovascular Imaging Research Core).

### Hypothetical Bypass Analysis

To reduce delays to MT, some have proposed direct emergency medical services routing to endovascular-capable centers.^12–14^ To evaluate the potential impact of such a strategy, we performed hypothetical bypass modeling for patients who had been brought by emergency medical services first to a nonendovascular hospital and then transferred by ground ambulance to the endovascular-capable center (air transfer patients were excluded because the distances can be farther and ground velocity could not be predicted). The projected direct-to-endovascular-capable hospital (bypass) time was calculated based on the actual driving distance from the scene directly to the endovascular-capable center combined with the actual average ambulance velocity observed during the interfacility transport and a projected average emergency medical services time on scene of 15 minutes.^15–17^ For patients with IV-tPA, the average (mean) door–to–tissue-type plasminogen activator (tPA) time at endovascular-capable sites was then added to predict onset-to-tPA time. Any patients whose predicted bypass onset-to-tPA time exceeded guideline recommendations^15^ were identified as no longer tPA-eligible. Finally, average door-to-puncture time was added to yield total bypass-projected treatment time. This analysis was repeated for patients initially within 20 miles of the endovascular-capable center because nearer bypasses may be more plausible.

### Statistical Analysis

Standard descriptive statistics were used, including mean, SD, and median with interquartile range (IQR) for continuous variables and frequency distributions for categorical variables. For between-group comparisons, *t* tests and Wilcoxon’s rank sum were used for continuous variables, and χ^2^ tests and Fisher exact tests was used for categorical variables. Two-tailed *P* values <0.05 were considered statistically significant. Statistical analyses were conducted in SAS version 9.2 or above (SAS Institute) and R version 3.2 or above (R Foundation for Statistical Computing). Outcome variables (mRS) were modeled against predictors of interest using logistic regression adjusted for age, qualifying NIHSS, occlusion location, use of tPA, transfer status, and time from onset. In this model, age, NIHSS, and time from onset were regarded as continuous predictors, whereas occlusion location, tPA, and transfer status were categorical. Time from onset, as the continuous predictor of principal interest, was also evaluated using a quadratic term in the regression. Confidence intervals (CIs) for odds ratios (ORs) were derived from these models using normal approximation based on standard errors, whereas predicted probabilities from the same models are presented in graphical format. The distribution of mRS between groups was compared using the Mantel-Haenszel ordinal χ^2^ statistic.

Additionally, as a sensitivity analysis, propensity scores were constructed with transfer status as the object of propensity scoring and age, qualifying NIHSS, vessel of target occlusion, tPA administration, ASPECTS score at baseline, sex, arterial occlusion location, smoking status, history of atrial fibrillation, diabetes mellitus, history of recent stroke, carotid artery stenosis, history of coronary disease, hyperlipidemia, prestroke functional status, and hypertension as predictors. These covariates reflect all the variables analyzed in the recent randomized trials of endovascular treatment for stroke^4–8^ and the subsequent meta-analyses.^18,19^ The model for outcome (functional independence, mRS 0–2) was then constructed using transfer status, time from onset to arterial puncture, and propensity as predictors in 2 fashions: by using the propensity score as a covariate in the model, and by stratification into 5 groups based on propensity scores as per Rosenbaum and Rubin.^20^

## Results

A total of 1000 patients were enrolled over 22 months, but 16 did not meet the inclusion criteria (5 had baseline mRS>2, 4 were treated beyond 8 hours, 2 consented outside the enrollment window, 2 were without LVO, 1 lacked documentation of consent, 1 had an NIHSS<8, and 1 had missing data), leaving 984 patients available for analysis. Direct presentation to the endovascular-capable site was observed in 539 patients, and the remaining 445 were transferred from another hospital to the endovascular-capable hospital for treatment. Baseline characteristics were mostly similar between these 2 groups (Table 1), with predictable differences in mean initial NIHSS score (18.0±5.5 for transfers versus 16.7±5.5 for direct; *P*=0.0006) and ASPECTS score by core laboratory assessment (7.9±1.8 for transfers versus 8.4±1.4 for direct; *P*<0.0001). IV-tPA was administered to 628 patients: 329/539 (61.0%) of direct patients and 299/445 (67.2%) of transferred patients (*P*=0.044).

**Table 1.**
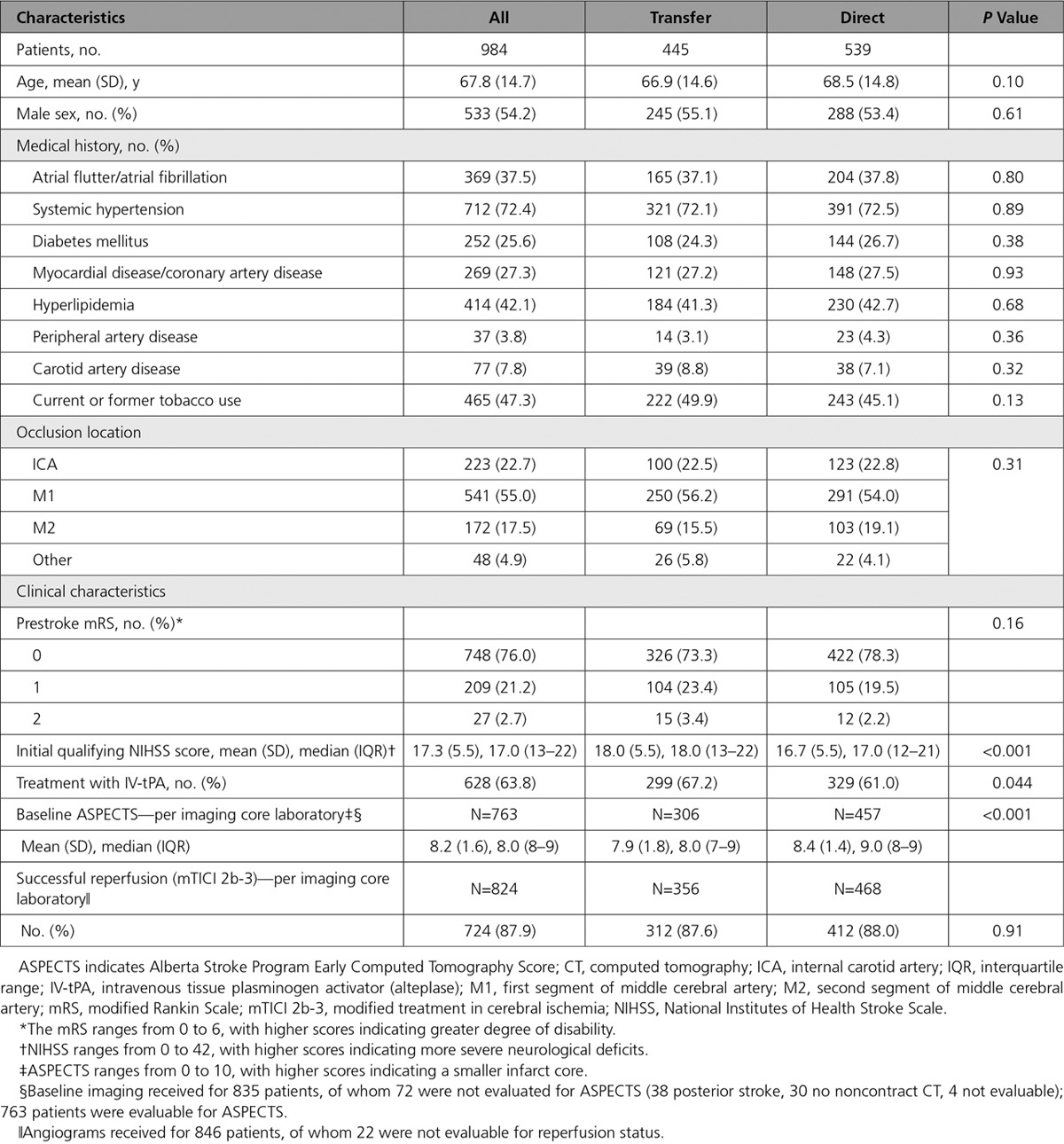
Baseline and Clinical Characteristics According to Admission Status

### Time Metrics

**Figure 1.**
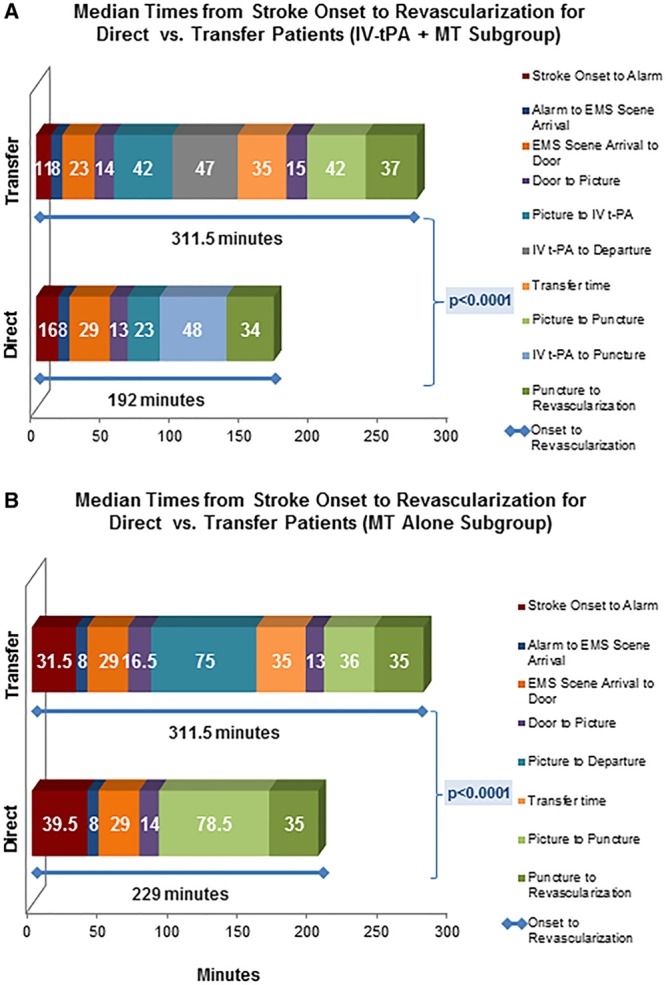
**Median time intervals from stroke onset (the time of last seen well) through revascularization. A**, All patients who received IV-tPA before MT. There is a significant difference in onset-to-revascularization times (blue line). **B**, All patients who underwent MT alone (no IV-tPA). There is a significant difference in onset-to-revascularization times (blue line). EMS indicates emergency medical services; IV-tPA, intravenous tissue plasminogen activator; and MT, mechanical thrombectomy.

Median onset-to-revascularization time was 202.0 minutes (IQR, 160–265) in the direct group compared with 311.5 minutes (IQR, 255–386) in the transfer group (*P*<0.001), with a difference between the mean times of 100 minutes. The mean alarm-to-revascularization time, measuring system efficiency defined as time of 911 call to treatment reperfusion, differed by 116 minutes. Among patients who received tPA, median onset-to-revascularization time was 192.0 minutes (IQR, 156–239) for the direct group compared with 311.5 minutes (IQR, 257–381) for the transferred group (*P*<0.001). A comparison of mean times yields a difference of 124 minutes between the 2 groups, which was primarily related to longer door-to-tPA times at nonenrolling hospitals (median, 54.5 versus 37.0, *P*<0.001), delays between IV-tPA and departure from the initial hospital (median, 47 minutes; IQR, 27–85), and length of transport time (median, 35 minutes; IQR, 21–58) (Figure 1A). Similarly among MT-alone patients, median onset-to-revascularization time was 229.0 minutes (IQR, 175–363) for the direct group compared with 311.5 minutes (IQR, 247–401) for the transferred group (*P*<0.001), which was primarily related to the delay between imaging completion and departure from the transferring hospital (median, 75 minutes; IQR, 55–116) plus the length of transport time (median, 35 minutes; IQR, 23–50) (Figure 1B).

### Clinical Outcomes

**Figure 2.**
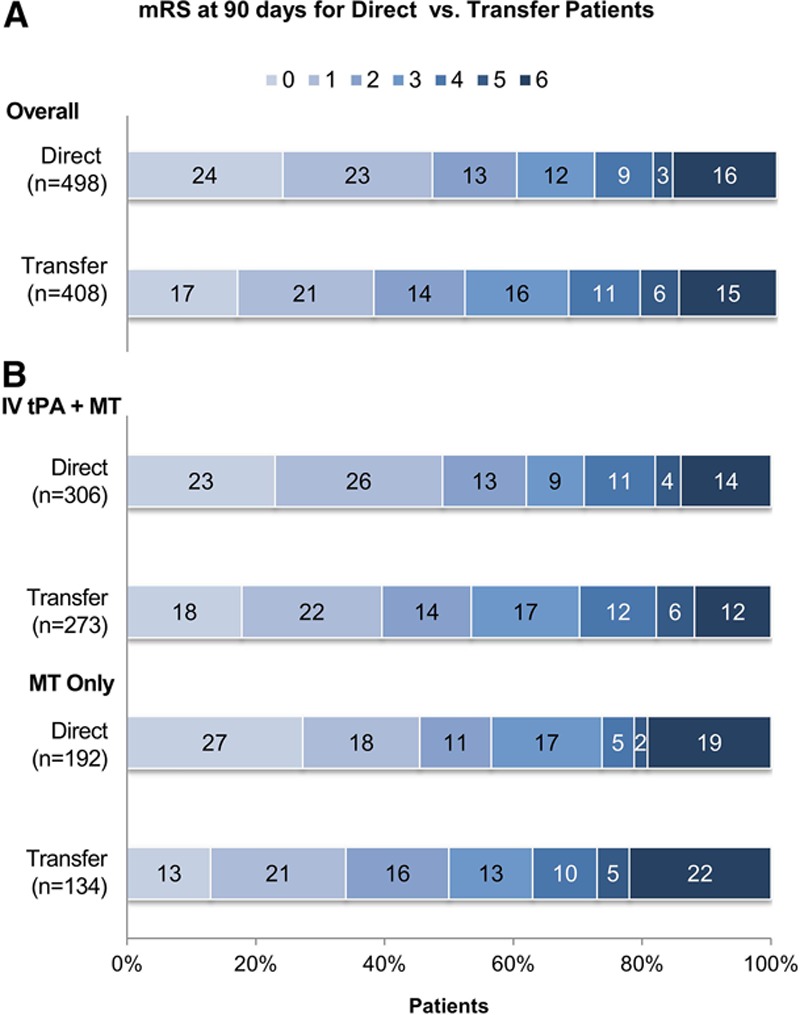
**Unadjusted clinical outcomes at 90 days based on mRS, presented as percentage of the total. A**, All patients, divided by direct admission (**top**) vs. interhospital transfer (**bottom**). There is a significant difference between the 2 groups by shift analysis (*P*=0.012 by Cochran-Mantel-Haenszel test). **B**, Comparison of outcomes based on mRS between direct and transfer divided into patients who received IV-tPA before MT (**top**) and those who underwent MT alone (**bottom**). Shift analysis revealed a significant difference between transfer and direct groups for MT alone (*P*=0.035) and a nonsignificant trend for IV-tPA (*P*=0.14). IV-tPA indicates intravenous tissue plasminogen activator; mRS, modified Rankin Scale; and MT, mechanical thrombectomy.

Clinical outcomes at 90 days were better in the direct group, with 60.0% (299/498) achieving functional independence (mRS 0–2) compared with 52.2% (213/408) in the transfer group (unadjusted OR, 1.38; 95% CI, 1.06–1.79; *P*=0.02) (Figure 2A). Likewise, excellent outcome of mRS 0 or 1 was achieved in 47.4% (236/498) of direct patients versus 38.0% (155/408) of transfer patients (OR, 1.47; 95% CI, 1.13–1.92; *P*=0.005). Comparing overall outcome by mRS shift analysis also favored direct presentation (*P*=0.012 by Cochran-Mantel-Haenszel test). Mortality did not differ between the 2 groups (15.0% for direct, 13.7% for transfer; *P*=0.56).

Among patients who received IV-tPA before MT, outcomes at 90 days were better in the direct group, with 62.1% (190/306) achieving mRS 0 to 2 compared with 53.5% (146/273) in the transfer group (OR, 1.42; 95% CI, 1.02–1.98; *P*=0.036) (Figure 2B). Excellent outcomes (mRS 0–1) for patients who received IV-tPA before MT were also observed more frequently in the direct group compared with the transfer group (48.7% versus 39.9%; OR, 1.43; 95% CI, 1.03–1.99; *P*=0.04). In the MT-alone group, functional independence was more common in the direct group (56.8%; 109/192) versus the transfer group (50.0%; 67/134), although this difference was not statistically significant (*P*=0.23) (Figure 2B). For MT alone, excellent outcomes (mRS 0–1) were also observed more frequently in the direct group compared with the transfer group (45.3% versus 34.3%; OR, 1.59; 95% CI, 1.00–2.50; *P*=0.047). Comparison of outcomes by mRS shift analysis revealed a significant difference between transfer and direct groups for MT alone (*P*=0.035) and a nonsignificant trend for IV-tPA (*P*=0.14).

**Figure 3.**
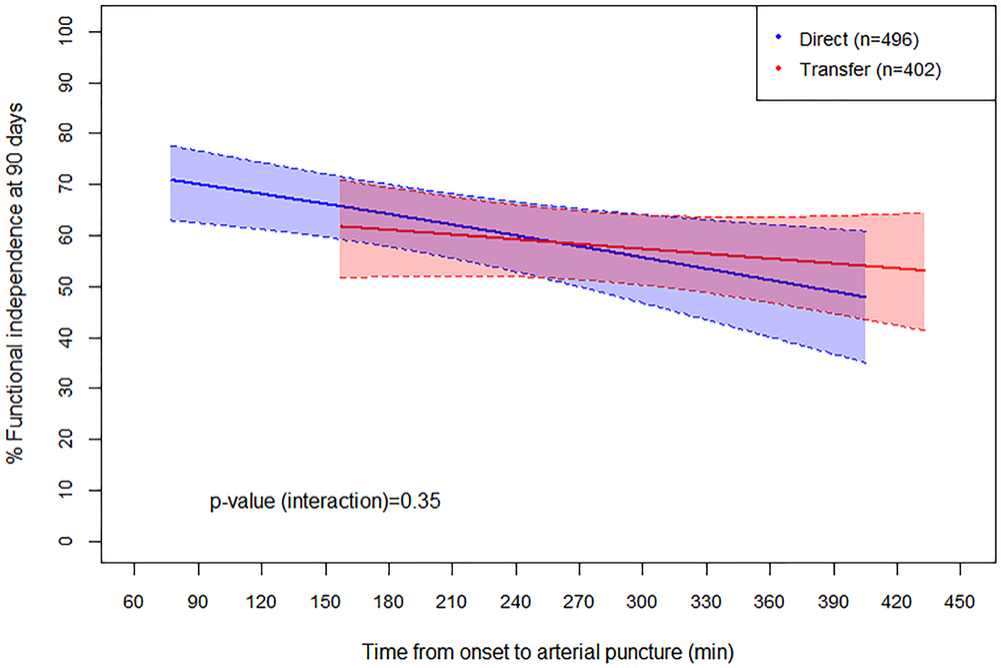
**Relationship between rate of functional independence (mRS 0–2 at 90 days) and time from onset to puncture for direct (blue) vs. transfer (red) patients.** The logistic curves have been truncated at the 95% distribution for each group, and thus the transfer group is shifted to the right (later average treatment time) compared with the direct group. Shading represents the 95% confidence interval for each group. The slopes do not differ between the 2 groups (*P*=0.35), suggesting that differences in outcome are related only to time. The rate of functional independence decreased by 5.5% per hour for all patients. mRS indicates modified Rankin Scale.

To determine the effect on outcome attributable to delay in time to treatment, multivariate logistic regression was used with the outcome of functional independence (mRS 0–2) by time from onset while accounting for multiple covariates (Table 2). Good outcome was less likely with increasing age, increasing NIHSS before treatment, and intracranial internal carotid artery occlusion (versus middle cerebral artery). Increasing time from onset to treatment was also significantly associated with lower likelihood of good outcome, with an adjusted OR of 0.93 for every additional 30-minute delay (95% CI, 0.89–0.98; *P*=0.008). When time to treatment was accounted for, there was no additional effect of direct presentation versus transfer on functional outcome (adjusted OR, 1.05; 95% CI, 0.76–1.45). Figure 3 displays the effect of time to treatment on outcome for both direct and transfer patients. Although time to treatment is systematically longer in the transfer group, the effect of time on outcome as measured by the slopes of the 2 response curves is similar (*P*=0.35, test of interaction effect between transfer status and time), supporting the hypothesis that the difference between the 2 groups is solely because of treatment delay. Across all patients, the absolute rate of functional independence decreased by 5.5% per hour from alarm to puncture. Additional univariate analysis showed that successful reperfusion was also strongly correlated with functional independence (OR, 2.21; 95% CI, 1.42–3.43).

**Table 2.**
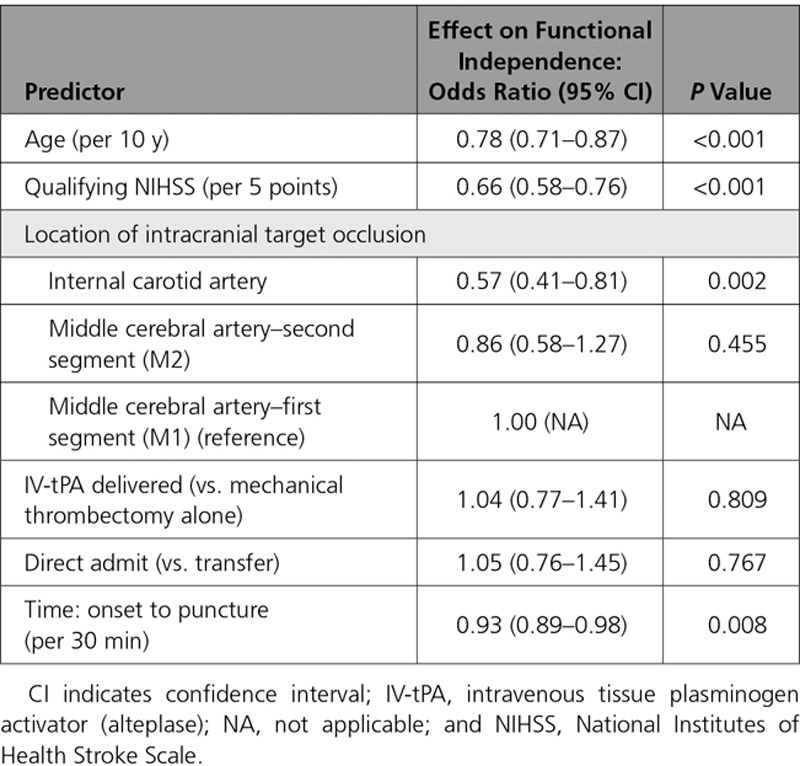
Effect on Good Functional Outcome Attributable to Delay in Time to Treatment

We performed an additional propensity score analysis to assess the role of all available standard stroke covariates, including age, qualifying NIHSS, vessel of target occlusion, tPA administration, ASPECTS score at baseline, sex, arterial occlusion location, smoking status, history of atrial fibrillation, diabetes mellitus, history of recent stroke, carotid artery stenosis, history of coronary disease, hyperlipidemia, prestroke functional status, and hypertension. After further adjustment for these variables and as seen in the multivariate regression analysis, results after propensity adjustment indicated a significant effect of time but not transfer status, as shown in Table 3. Also as before, the interaction between tPA and time was nonsignificant in the propensity-adjusted models, with *P*=0.14 for the covariate model and *P*=0.15 for the stratified model.

**Table 3.**
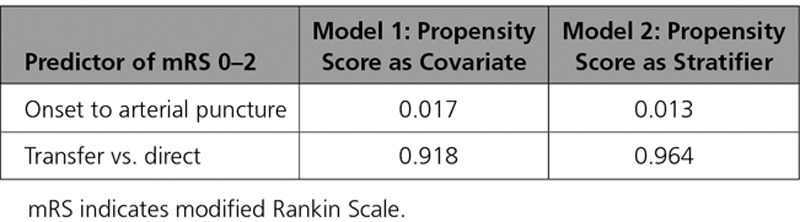
Predictors of mRS 0 to 2 at 90 Days Using Propensity Score Analysis

### Hypothetical Bypass Analysis

A total of 209 patients were transferred via ground and available for analysis in the hypothetical bypass scenario. Of these patients, 122 (58.4%) received IV-tPA at the first hospital, with a median onset-to-tPA time of 106 minutes (IQR, 81–136). Using the bypass model, 116 (95.1%) would still receive IV-tPA (6 no longer met guideline recommendations), and the median onset-to-tPA time would increase to 123 minutes (IQR, 90–160). The median onset-to-puncture time in all 209 patients was 250 minutes (IQR, 201–330), which would decrease to 169 minutes (IQR, 133–223) with hypothetical bypass. When comparing mean times, tPA would be administered 12.0 minutes later (±SD of 46.9), but endovascular treatment would be delivered 91.0 minutes sooner (±47.6).

A total of 130 patients were transferred despite initially being within 20 miles of the endovascular hospital. Among these patients, 71 (54.6%) received IV-tPA at the first hospital, with a median onset-to-tPA of 100 minutes (IQR, 80–130). In the hypothetical bypass model, 69 (97.2%) would still receive IV-tPA (2 excluded by guidelines), and median onset-to-tPA would increase to 102 minutes (IQR, 78–143). Median onset-to-puncture in all 130 patients was 240 minutes (IQR, 190–310), which would decrease to 148 minutes (IQR, 117–198) with hypothetical bypass. When comparing mean times for the patients within 20 miles, tPA would be administered 6.9 minutes later (±SD of 44.8), but endovascular treatment would be delivered 94.0 minutes sooner (±46.4).

## Discussion

This study provides important information regarding the impact of systems of care for patients with LVO stroke undergoing MT. We found that interhospital transfer was associated with mean treatment delays of 116 minutes compared with direct presentation to the endovascular-capable center. Interhospital transfer was associated with lower chances of excellent clinical outcome (47.4% versus 38.0%) and lower chances of functional independence (60.0% versus 52.2%). The difference in rates of good outcome between the 2 groups was entirely attributable to time delays; when controlling for time from onset to treatment, there was no difference in outcomes between direct and transfer patients.

Time delays in the transfer group were mainly attributable to 3 epochs: picture to departure for MT alone (75 minutes), tPA to departure for tPA+MT (47 minutes), and transfer transport time (35 minutes). The first 2 time epochs suggest that much of the delay associated with transfer is because of medical decision making or the logistics of arranging transport. Despite these delays, other time epochs were actually shorter in the transfer group (such as picture-to-puncture times at the enrolling hospital), presumably because of advance notice of the patient’s admission.

The relationship between time to treatment and outcomes has been described in several recent studies. The recent HERMES analysis (Highly Effective Reperfusion Evaluated in Multiple Endovascular Stroke Trials) reported a linear reduction in chances of good outcome with increasing time between onset and MT, with no significant benefit beyond 7.3 hours.^10^ A subanalysis of the SWIFT PRIME study (Solitaire FR With the Intention for Thrombectomy as Primary Endovascular Treatment for Acute Ischemic Stroke) found a high rate of good outcomes for patients treated with MT within 2.5 hours of onset, but this rate decreased by 10% over the next hour and by 20% with every subsequent hour of delay.^21^ In the current study, we found that the rate of good outcome was reduced by 5.5% for each 1-hour delay from alarm to puncture. The difference between these 2 studies likely reflects selection bias: patients enrolled in the randomized study were selected by a strict set of inclusion criteria, whereas patients in this real-world registry were treated based on the opinion of the local treatment team. These treatment decisions were likely based on various factors predictive of outcome, and patients likely to have a good outcome despite longer time from onset were probably still treated. Furthermore, the accelerated reduction in chance of good outcome after 3.5 hours that was seen in SWIFT PRIME^21^ was not observed in the current study. This difference is also likely attributable to enrollment differences: patients who may have otherwise been eligible for MT but had imaging signs of established infarction were likely not treated as they were in SWIFT PRIME.

It is interesting to note that another recent but smaller registry of MT did not find a significant difference in outcome between direct and transfer patients, despite onset-to-recanalization times of 297 versus 240 minutes.^22^ However, that study was smaller (159 patients), included only a single referring center and a single MT center, and observed a smaller time difference between direct and transfer patients. Given that we observed a 5.5% reduction in good outcomes for each hour of delay, the results of this smaller study are not necessarily incongruent and may reflect circumstances specific to that singular center. Another recent study using a large national database reported a higher mortality rate associated with transfer (18.6%) compared with direct presentation (14.9%) for MT.^23^ Although our study did not reveal a mortality difference between the 2 groups, the overall mortality rates are similar between the 2 studies. Given that the database study included 8533 patients, it is certainly reasonable to think that a small but significant mortality difference does exist between direct and transfer patient cohorts.

The administration of IV-tPA did not have a significant impact on outcome in this cohort, and time to MT remained the most important determinant of outcome. However, it is important to note that any patient who successfully achieved thrombolysis with IV-tPA alone would not undergo MT and would become ineligible for the current registry. The impact of IV-tPA before MT was recently explored in a pooled analysis of the SWIFT and STAR studies (Solitaire FR With the Intention for Thrombectomy and Solitaire FR Thrombectomy for Acute Revascularization).^24^ That study showed a nonsignificant difference in rates of good outcome between patients treated with IV-tPA plus MT versus MT alone (57.7% versus 47.7%; *P*=0.10). Again, patients who responded to tPA before MT would not have been included. The true benefit of early tPA administration for LVO before MT can be assessed only with a properly conducted controlled trial, which is now clearly needed.

Currently, IV-tPA is often cited as the reason for avoiding longer transport to an endovascular-capable center, so that IV-tPA can be administered sooner at a nearer hospital.^25–27^ In the current study, analysis of all patients undergoing interhospital transfer by ground ambulance suggested that a direct-to-endovascular bypass would delay IV-tPA to a modest degree (12 minutes) and 5% would no longer be eligible for tPA, but the start of endovascular therapy would be greatly accelerated (91 minutes sooner). When limiting the hypothetical model only to patients initially within 20 miles of the endovascular center, bypass becomes even more appealing: tPA is delayed by only 6.9 minutes, only 3% miss the opportunity for tPA, and endovascular treatment starts 94 minutes sooner.

A significant delay to treatment because of interhospital transfer may also create a missed opportunity for MT. Although such patients would not be captured by the current registry, Sablot et al^28^ recently described a series of LVO patients transferred for MT after receiving IV-tPA at a nonendovascular center. Of 119 patients transferred, only 52 (44%) actually underwent MT. Significant treatment delays associated with interhospital transfer were identified as the primary reason that MT was withheld. Thus, although bypassing the closer hospital may create missed opportunities for IV-tPA, interhospital transfer appears to create significant missed opportunities for MT.

As systems of care change to accommodate and optimize acute stroke treatments, the triage and transport of patients with acute stroke suspected of having LVO may have a significant impact on the emergency department resources. Currently available clinical scales for use in the field have high rates of false-positive results, with <50% of suspected patients actually undergoing MT.^29^ Such an influx of unnecessary emergency department admissions may create a significant burden on treatment teams. Clearly, more accurate means of rapid triage and diagnosis are needed.

In the interest of accelerating time to treatment with both IV-tPA and MT, one might consider offering endovascular therapy at more hospitals (ie, those that are not currently endovascular-capable). In fact, a majority of designated Primary Stroke Centers already offer some form of endovascular treatment, despite not being designated as Comprehensive Stroke Centers.^30^ Conceptually, decentralizing the availability of neurointerventionalists would improve treatment times. However, others have shown that mortality after stroke intervention is clearly dependent on volume: 1 study reported mortality of 19.7% at low-volume, 14.9% at medium-volume, and 9.8% at high-volume centers (*P*=0.003).^31^ Although it is unclear whether these mortality differences are related to the procedure or perioperative care, some have argued for transportation of the neurointerventionalist to the patient, rather than the other way around. Hui et al^32^ reported a single case of LVO stroke where the neurointerventionalist was flown to a nonendovascular Primary Stroke Center and carried out MT there. Patient outcome and disposition were not reported, but the case does provide proof of concept and may justify further investigation.

This study does have several limitations. Some differences between the direct and transfer groups were observed. Specifically, transferred patients had higher initial NIHSS, suggesting patients with more severe strokes were transferred for MT. Aside from stroke severity as assessed by the NIHSS, other selection biases may have led to greater differences between the groups that could not be accounted for with regression analysis. In fact, additional confounders, such as time of day or insurance status, are unknown and unaccounted for in the current study. Furthermore, transferred patients were more often treated with tPA, which may be related to earlier presentation at the community hospital. The ASPECTS on imaging before MT was worse in the transfer group compared with the direct group, likely a product of longer delays to treatment in the transferred patients. Although these differences were accounted for by multivariate regression, they may still have an influence on the analysis. Another important limitation of this study is that patients who experienced successful recanalization with IV-tPA would be excluded. Finally, the hypothetical bypass analysis is potentially biased by unknown variables, such as situational traffic delays.

### Conclusions

This large, real-world registry of MT for stroke because of LVO has shown that interhospital transfer before endovascular treatment with MT is associated with delays to treatment and a significantly lower chance of good outcome compared with direct presentation to the endovascular-capable center. Strategies to facilitate more rapid identification of LVO and direct routing to endovascular-capable centers for patients with severe stroke may help improve outcomes.

## Sources of Funding

This study was supported by Medtronic.

## Disclosures

No authors received any payments for work on the submitted manuscript. Dr Froehler serves as a scientific consultant to Medtronic and Blockade and has received a National Institutes of Health grant. Drs Mueller and Zaidat serve as scientific consultants regarding trial design and conduct to Medtronic. The University of California, Regents receives funding for Dr Saver’s services as a scientific consultant regarding trial design and conduct to Covidien and Stryker and is an employee of the University of California, which holds a patent on retriever devices for stroke. Dr Sultan serves as a scientific consultant to Medtronic. The University of California, Regents receives funding for Dr Jahan’s services as a scientific consultant regarding trial design and conduct to Medtronic/Covidien and is an employee of the University of California, which holds a patent on retriever devices for stroke. Dr Klucznik is on the Speakers’ Bureau for Medtronic. Dr Hellinger is on the Speakers’ Bureau for Medtronic and serves as a consultant to Penumbra and Cordis Neurovascular (Johnson and Johnson). Dr Yavagal has received honoraria from Medtronic and serves as a scientific consultant to Medtronic and Neuralanalytics, Inc. Dr Yao serves as a consultant/proctor to Medtronic. Dr Liebeskind has received a National Institute of Health grant and serves as a scientific consultant to Stryker and Medtronic. Drs Abou-Chebl and Hassan have received honoraria from Medtronic. Dr Baxter is on the Spearkers’ Bureau, has received honoraria, has ownership interest, and serves as a consultant to Penumbra. Dr Bozorgchami serves as a consultant to Neuravi. Dr Delgado has received honoraria from Penumbra. Dr English has received honoraria from Medtronic, Stryker Neurovascular, and Penumbra. Dr Given is on the Speakers’ Bureau and serves as a consultant to Medtronic. Dr Gupta serves as a consultant to Medtronic and Stryker Neurovascular. Dr Linfante is on the Speakers’ Bureau for Medtronic and Stryker and has ownership interest in Three Rivers. Dr Nogueira serves as a consultant to Medtronic, Stryker Neurovascular, Penumbra, Neuravi, Allm Inc. Dr Schirmer has received honoraria from the American Association of Neurological Surgeons and Toshiba and has ownership interest in NTI. Dr Siddiqui serves as a consultant to Medtronic, Codman, Penumbra, Stryker, Microvention, Guidepoint Global Consulting, WL Gore & Associates, Three Rivers, Corindus, Amnis Therapeutics, CereVasc, Pulsar Vascular, The Stroke Project, Cerebrotech Medical Systems, Rapid Medical, Neuravi, Silk Road Medical, Rebound Therapeutics, Claret Medical, Intersocietal Accreditation Commission, and Medical University of South Carolina and has ownership interest in StimMed, Valor Medical, Cardinal Health, and Medina Medical Systems. Dr Taqi serves as a consultant to Stryker Neurovascular.

## Supplementary Material
