# Supplementary material for: Interhospital Transfer Before Thrombectomy Is Associated With Delayed Treatment and Worse Outcome in the STRATIS Registry (Systematic Evaluation of Patients Treated With Neurothrombectomy Devices for Acute Ischemic Stroke)
Source: Circulation. 2017 Dec 11;136(24):2311–21. doi: 10.1161/CIRCULATIONAHA.117.028920 (PMC5732640; doi:10.1161/CIRCULATIONAHA.117.028920)
Supplement: Supplementary file 1 [file cir-136-2311-s001.pdf]

SUPPLEMENTAL MATERIAL

Interhospital transfer prior to thrombectomy is associated with delayed treatment and worse outcome in the STRATIS registry

Supplemental Table I. STRATIS Hospital Characteristics

Site #	Hospital Name	City	State	Geographic Type[a]	Stroke Center Type[b]	Certifying Body[b]	STRATIS enrollment direct	STRATIS enrollment transfer	STRATIS total enrollment	# Beds[a]	Volume of tPA 2015[a]	Volume of MT Procedures 2015[a]
101	OHSU Marquam Hill Campus	Portland	OR	Urban	Comprehensive	Joint Commission	5	28	33	564	14	37
102	Ruby Memorial Hospital	Morgantown	WV	Urban	Primary	Joint Commission	6	0	6	455	89	29
103	Jackson Memorial Hospital	Miami	FL	Urban	Comprehensive	Joint Commission	48	10	58	1450	75	55
104	Baptist Health Lexington	Lexington	KY	Urban	Primary	Joint Commission	28	5	33	360	47	77
105	Maine Medical Center	Portland	ME	Urban	Primary	Joint Commission	10	6	16	611	43	58
106	Cleveland Clinic	Cleveland	OH	Urban	Comprehensive	Joint Commission	2	15	17	1258	45	70
107	Providence St Vincent Medical Center	Portland	OR	Urban	Comprehensive	Joint Commission	15	11	26	526	56	25
108	Presence St. Joseph Medical Center/ Presence Resurrection Medical Center	Chicago	IL	Urban	Primary	Joint Commission	6	5	11	432 / 200	27 / 13	31/24
109	Brigham and Women's Hospital	Boston	MA	Urban	Primary	State of Mass	5	26	31	763	13	27
110	Buffalo General Medical Center	Buffalo	NY	Urban	Comprehensive	Det Norske Veritas (DNV)	6	0	6	942	113	111
111	Saint Lukes Hospital of Kansas City	Kansas City	MO	Urban	Comprehensive	Joint Commission	14	21	35	387	33	111
112	Baptist Medical Center Jacksonville	Jacksonville	FL	Urban	Primary	Joint Commission	6	7	13	867	48	85
113	Grady Memorial Hospital	Atlanta	GA	Urban	Comprehensive	Joint Commission	20	44	64	639	68	346
114	Florida Hospital Orlando	Orlando	FL	Urban	Primary	Det Norske Veritas (DNV)	46	17	63	2393	132	127
115	Memorial Hermann-Texas Medical Center	Houston	TX	Urban	Comprehensive	Joint Commission	5	3	8	931	151	49
116	WellStar Kennestone Regional Medical Center	Marietta	GA	Urban	Comprehensive	Joint Commission	38	15	53	552	92	156

Site #	Hospital Name	City	State	Geographic Type[a]	Stroke Center Type[b]	Certifying Body[b]	STRATIS enrollment direct	STRATIS enrollment transfer	STRATIS total enrollment	# Beds[a]	Volume of tPA 2015[a]	Volume of MT Procedures 2015[a]
117	Crouse Hospital	Syracuse	NY	Urban	Primary	State of New York	4	0	4	487	52	67
118	Ohio Health Research Institute	Columbus	OH	Urban	Comprehensive	Joint Commission	6	9	15	671	35	78
119	Advocate Christ Medical Center / Advocate Lutheran General Hospital	Chicago	IL	Urban	Primary	Det Norske Veritas (DNV)	5	8	13	580 / 530	27 / 31	41 / 43
120	University of Massachusetts Medical Hospital	Worcester	MA	Urban	Primary	State of Mass	4	1	5	572	56	35
121	Mercy San Juan Medical Center	Sacramento	CA	Urban	Comprehensive	Joint Commission	4	5	9	370	65	105
122	Houston Methodist Hospital	Houston	TX	Urban	Comprehensive	Det Norske Veritas (DNV)	11	2	13	914	63	46
123	Palmetto General Hospital/ Delray Medical Center/ St. Mary's Medical Center	Hialeah/ Delray Beach/ West Palm Beach	FL	Urban	Comprehensive	Joint Commission	53	18	71	360 / 347 / 374	61 / 52 / 101	63 / 63 / 12 / 82
125	Froedtert Hospital	Milwaukee	WI	Urban	Comprehensive	Joint Commission	2	2	4	508	30	54
126	Norton Hospital	Louisville	KY	Urban	Comprehensive	Joint Commission	26	31	57	1362	34	118
127	Barnes - Jewish Hospital South	St. Louis	MO	Urban	Comprehensive	Joint Commission	3	8	11	1206	63	994
128	Valley Baptist Medical Center	Harlingen	TX	Urban	Primary	Det Norske Veritas (DNV)	18	21	39	378	28	111
129	Ascension St John Hospital/ Providence Hospital and Medical Center	Detroit / Southfield	MI	Urban	Primary	Joint Commission	1	2	3	573 / 575	34 / 29	20 / 25
130	Aurora St Lukes Medical Center	Milwaukee	WI	Urban	Comprehensive	Joint Commission	0	7	7	882	53	33
131	University Of Kentucky Hospital - Albert B Chandler Hospital	Lexington	KY	Urban	Comprehensive	Joint Commission	3	4	7	829	58	56
132	Memorial Regional Hospital	Hollywood	FL	Urban	Primary	Joint Commission	29	1	30	909	31	46

Site #	Hospital Name	City	State	Geographic Type[a]	Stroke Center Type[b]	Certifying Body[b]	STRATIS enrollment direct	STRATIS enrollment transfer	STRATIS total enrollment	# Beds[a]	Volume of tPA 2015[a]	Volume of MT Procedures 2015[a]
133	Ronald Reagan UCLA Medical Center	Los Angeles	CA	Urban	Comprehensive	Joint Commission	13	8	21	452	21	32
134	Banner University Medical Center Tucson	Tucson	AZ	Urban	Primary	Joint Commission	6	2	8	479	24	<11
135	McLaren Flint	Flint	MI	Urban	Comprehensive	Joint Commission	1	3	4	284	40	18
136	Virginia Mason Medical Center	Seattle	WA	Urban	Primary	Joint Commission	1	2	3	236	<11	<11
137	Geisinger Medical Center	Danville	PA	Urban	Primary	Joint Commission	4	5	9	504	37	13
138	St. Dominic - Jackson Memorial Hospital	Jackson	MS	Urban	Comprehensive	Joint Commission	10	8	18	535	44	47
139	Baylor University Medical Center	Dallas	TX	Urban	Comprehensive	Det Norske Veritas (DNV)	8	6	14	850	55	22
141	University of Maryland Medical Center	Baltimore	MD	Urban	Comprehensive	Joint Commission	1	8	9	711	20	33
142	ProMedica Toledo Hospital	Toledo	OH	Urban	Comprehensive	Joint Commission	0	2	2	654	30	85
143	Baptist Hospital of Miami	Miami	FL	Urban	Comprehensive	Joint Commission	9	6	15	705	68	63
144	Los Robles Hospital and Medical Center	Thousand Oaks	CA	Urban	Primary	Det Norske Veritas (DNV)	3	1	4	294	11	77
145	Baptist Health Louisville	Louisville	KY	Urban	Primary	Joint Commission	8	2	10	468	48	18
146	Albany Medical Center	Albany	NY	Urban	Primary	Joint Commission	2	3	5	683	17	61
147	Swedish Medical Center - Cherry Hill	Seattle	WA	Urban	Primary	Det Norske Veritas (DNV)	1	12	13	170	34	53
148	Abbott Northwestern Hospital	Minneapolis	MN	Urban	Comprehensive	Det Norske Veritas (DNV)	1	0	1	579	37	44
149	Erlanger Baroness Hospital	Chattanooga	TN	Urban	Comprehensive	Joint Commission	2	0	2	606	87	182
150	University of California, Irvine Medical Center	Irvine	CA	Urban	Comprehensive	Joint Commission	3	0	3	349	56	26

Site #	Hospital Name	City	State	Geographic Type[a]	Stroke Center Type[b]	Certifying Body[b]	STRATIS enrollment direct	STRATIS enrollment transfer	STRATIS total enrollment	# Beds[a]	Volume of tPA 2015[a]	Volume of MT Procedures 2015[a]
151	California Pacific Medical Center - Davies Campus Hospital	San Francisco	CA	Urban	Primary	Joint Commission	0	1	1	146	<11	93
152	University of Tennessee Medical Center	Knoxville	TN	Urban	Comprehensive	Joint Commission	8	6	14	599	36	42
154	UPMC Presbyterian ShadySide	Pittsburgh	PA	Urban	Comprehensive	Joint Commission	24	26	50	1173	38	167
155	Vanderbilt University Medical Center	Nashville	TN	Urban	Comprehensive	Joint Commission	3	5	8	890	59	70
157	Banner - University Medical Center Phoenix	Phoenix	AZ	Urban	Primary	Joint Commission	1	2	3	643	47	51
158	Mayo Clinic Hospital	Rochester	MN	Urban	Comprehensive	Joint Commission	1	4	5	1079	18	48
159	Carolinas HealthCare - Carolinas Medical Center	Charlotte	NC	Urban	Primary	Joint Commission	0	1	1	1030	44	25

[a]Source: Definitive Healthcare-based on data from all payers in 2015: Volume of tPA is defined as stroke patients who received tPA with no further treatment for stroke (ICD9 code 43300, 43301, 43310, 43311, 43320, 43321, 43330, 43331, 43380, 43381, 43390, 43391,43400, 43401, 43410, 43411, 43490, 43491, ICD10 163.x, 166.x and DRG 061,062,063); Volume of MT procedures (ICD9 Procedure code 3974)

[b]Stroke center type information obtained from the following:

1. DNV Healthcare Introduces Comprehensive Stroke Center Certification. DNV GL Healthcare. http://dnvglhealthcare.com/releases/dnv-healthcare-introduces-comprehensive-stroke-center-certification. Accessed January 30, 2017.
2. The Joint Commission Quality Check. The Joint Commission. https://www.qualitycheck.org/data-download/certification-data-download/. Accessed January 30, 2017.
3. New York State Department of Health, NYS Health Profiles. https://profiles.health.ny.gov/hospital/designated_centers/Stroke+Center. Accessed January 30, 2017.
4. Commonwealth of Massachusetts, Executive Office of Health and Human Service. http://www.mass.gov/eohhs/gov/departments/dph/programs/hcq/healthcare-quality/health-care-facilities/hospitals/stroke-services/designated-primary-stroke-services-hospitals.html. Accessed January 30, 2017.
